# Clinical development of phosphatidylinositol 3-kinase inhibitors for cancer treatment

**DOI:** 10.1186/1741-7015-10-161

**Published:** 2012-12-11

**Authors:** Irene Brana, Lillian L Siu

**Affiliations:** 1 Drug Development Program, Division of Medical Oncology and Hematology, Princess Margaret Cancer Centre, University of Toronto, 610 University Avenue, Toronto, Ontario, M5G 2M9, Canada

**Keywords:** PI3K, isoform, neoplasm, patient selection, clinical trials, cancer

## Abstract

The phosphatidylinositol 3-kinase (PI3K) pathway is commonly deregulated in cancer. In recent years, the results of the first phase I clinical trials with PI3K inhibitors have become available. In comparison to other targeted agents such v-raf murine sarcoma viral oncogene homolog B1 (BRAF) inhibitors in melanoma or crizotinib in anaplastic lymphoma receptor tyrosine kinase (ALK) translocated tumors, the number of objective responses to PI3K inhibitors is less dramatic. In this review we propose possible strategies to optimize the clinical development of PI3K inhibitors: by exploring the potential role of PI3K isoform-specific inhibitors in improving the therapeutic index, molecular characterization as a basis for patient selection, and the relevance of performing serial tumor biopsies to understand the associated mechanisms of drug resistance. The main focus of this review will be on PI3K isoform-specific inhibitors by describing the functions of different PI3K isoforms, the preclinical activity of selective PI3K isoform-specific inhibitors and the early clinical data of these compounds.

## Introduction

**Figure 1:**
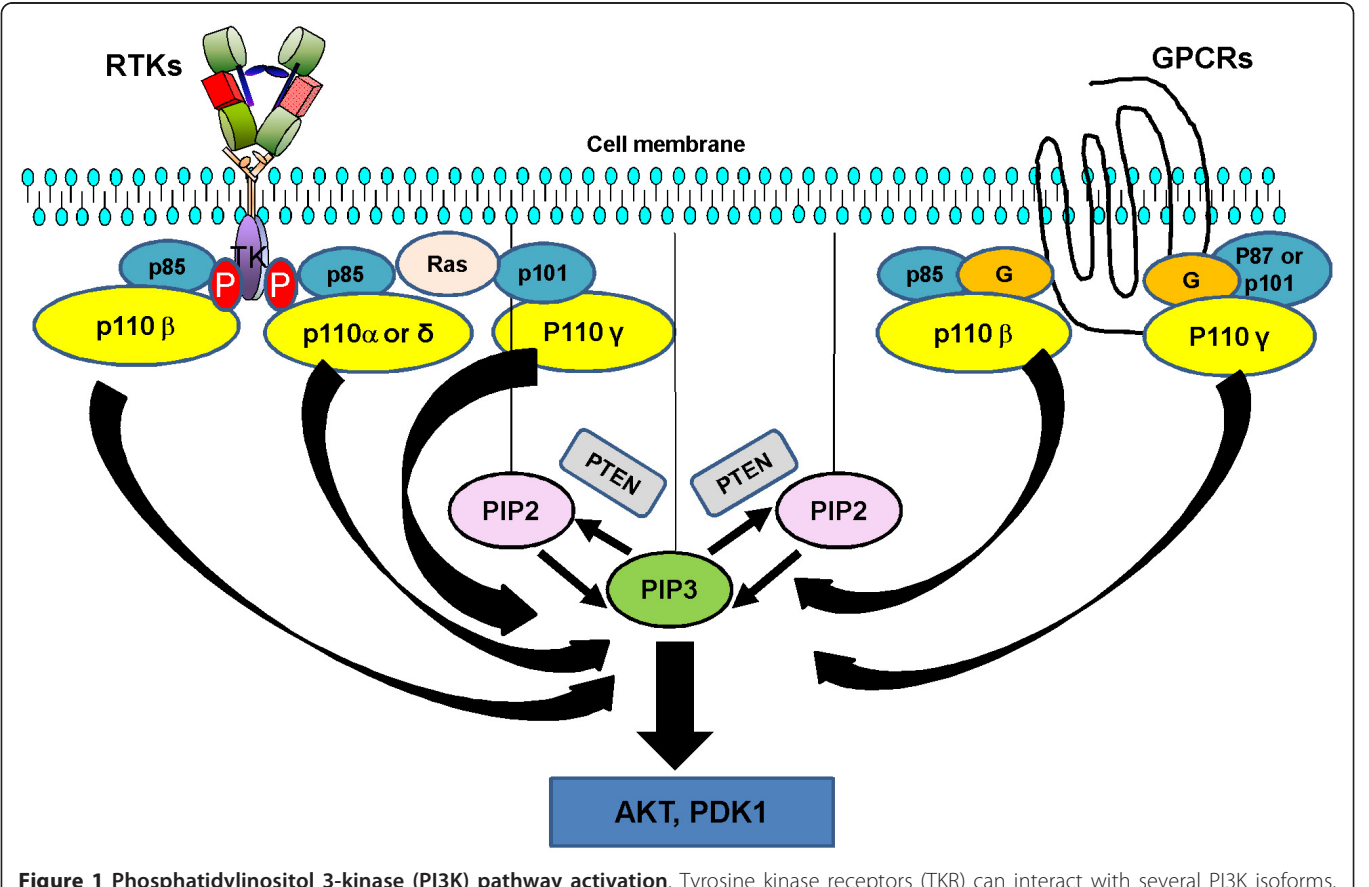
**Phosphatidylinositol 3-kinase (PI3K) pathway activation**. Tyrosine kinase receptors (TKR) can interact with several PI3K isoforms. RAS proteins can activate PI3Kα and γ isoforms. In addition certain RAS proteins can activate PI3Kδ isoform. G protein-coupled receptors (GPCR) preferentially interact with the PI3Kβ or γ isoforms. Once activated by any of these mechanisms, PI3K interacts with the lipid membrane phosphorylating phosphatidylinositol 4,5-bisphosphate (PIP2) generating phosphatidylinositol 3,4,5-trisphosphate (PIP3). PTEN (phosphatase and tensin homologue deleted on chromosome 10) converts PIP3 into PIP2, regulating the final amount of PIP3 generated. PIP3 triggers a signaling cascade through the activation of AKT, phosphoinositide-dependent protein kinase 1 (PDK1) and their downstream effectors. G = G protein G; p110 = PI3K catalytic subunit; p85 = class IA PI3K regulatory subunit; p87 = class IB PI3K regulatory subunit; p101 = class IB regulatory subunit.

Phosphatidylinositol 3-kinases (PI3Ks) represent a family of lipid kinases that plays a key role in signal transduction, cell metabolism and survival [1,2]. The PI3K family is divided into three classes, I, II and III, based on their substrate specificity and structure. Among them, class I PI3K seems to be the most relevant in cancer. Class I PI3K has a catalytic subunit (p110) and a regulatory subunit (p85) that stabilizes p110 and inactivates its kinase activity at basal state. Physiologically, PI3K transduces signals received from activated tyrosine kinase receptors (RTK), G protein-coupled receptors (GPCR) or from activated RAS. Upon receipt of such signals, the p85 regulatory subunit interacts with the phosphorylated tyrosine residues of activated RTKs. This engagement then causes release of the p85-mediated inhibition of p110, such that p110 can interact with the lipid membranes to phosphorylate phosphatidylinositol 4,5-bisphosphate (PIP2) to phosphatidylinositol 3,4,5-trisphosphate (PIP3). This reaction triggers a signaling cascade through the activation of AKT and its downstream effectors. The amount of PIP3 generated and resultant PI3K pathway activation are tightly regulated by the tumor suppressor protein, phosphatase and tensin homologue deleted on chromosome 10 (PTEN). PTEN can inactivate the PI3K pathway by converting PIP3 into PIP2 (Figure 1). The PI3K pathway can be activated not only via RTKs, but also by RAS and GPCR. RAS can activate the PI3K pathway by its direct interaction with p110α, p110γ, and p110δ subunits, while GPCRs can interact with p110β and p110γ subunits [2].

The PI3K pathway is commonly deregulated in cancer, with the most common events being mutation or increased gene copy numbers of *PIK3CA *or other *PI3K *isoforms, loss of expression of the pathway suppressors (for example, PTEN), or hyperactivation of RTKs through receptor overexpression or activating mutations (Table 1). The first results of several early phase I clinical trials investigating different PI3K inhibitors (Table 2) have been presented in recent years (Table 3). Other targeted agents evaluated in specific oncogenically addicted patient populations in the early trial setting, such as vemurafenib [3] or dabrafenib [4] in v-raf murine sarcoma viral oncogene homolog B1 (*BRAF*) V600E mutant melanoma, or crizotinib in echinoderm microtubule-associated protein-like 4-anaplastic lymphoma kinase (*EML4-ALK*) translocated non-small cell lung cancer [5], have demonstrated dramatic antitumor activity. In contrast, the objective responses observed thus far with PI3K inhibitors have been more modest and in many cases of short duration. Several strategies may be considered to optimize the development of PI3K inhibitors in clinical trials.

**Table 1:** Common alterations in phosphatidylinositol 3-kinase p110α isoform gene (PIK3CA), PIK3CB and phosphatase and tensin homologue deleted on chromosome 10 (PTEN) in cancer

Alteration	Occurrence (%)	References
PIK3CA mutations:		
Breast	26% (1,559/6,110)	[108]
Endometrium	24% (282/1,194)	[108]
Penis	29% (8/28)	[108,109]
Urinary tract	20% (189/942)	[108]
Large intestine	12% (779/6,710)	[108]
Stomach	12% (96/824)	[108]
Ovary	10% (163/1,590)	[108]
Cervix	10% (25/256)	[108]
PIK3CA amplifications:		
Gastric	67% to 36%	[110,111]
Papillary thyroid cancer	53% (265/499)	[112]
Head and neck	55% to 37%	[113,114]
Non-small cell lung cancer	31%	[115]
Squamous cell carcinoma	59% (31/52)	[116]
Cervical	70% to 44%	[117,118]
Ovarian	35% (54/152)	[119]
Prostate	28% (9/32)	[120]
Endometrial	12% to 15%	[121,122]
Breast	8% (8/92)	[123]
Triple negative	31%	[124]
Chronic lymphocitic leukemia	5%	[125]
PIK3CB amplification:		
Breast	5%	[72]
Non-small cell lung cancer squamous cell carcinoma	56%	[116]
PTEN loss of heterozygosity:		
Glioblastoma	59%	[126]
Prostate	15% to 70%	[127-130]
Breast	11% to 38%	[131,132]
Melanoma	33% (7/21)	[133]
Gastric	47% (14/30)	[111]
Uveal melanoma	76% to 39%	[134]
PTEN mutation:		
Endometrium	37% (690/1,860)	[135]
Vulva	62% (5/8)	[136]
Central nervous system	24% (491/2,055)	[137]
Prostate	14% (92/658)	[135]
Melanoma	16% (104/652)	[138]
Uveal melanoma	11% (4/35)	[134]

**Table 2:** Isoform specificity of some of the phosphatidylinositol 3-kinase (PI3K) inhibitors in clinical development

IC50 (nM)	α	E545K	H1047R	β	δ	γ	mTOR	Reference
Pan-isoform PI3K inhibitors								
XL147	39	-	-	383	36	23	> 15,000	[6]
BKM120	52	99	58	166	116	262	2,866	[139]
GDC-0941	3	3	3	33	3	75	580	[140]
PX-866	39	-	-	88	124	183	-	[13]
BAY 80-6946	0.5	-	-	3.7	0.7	6.4	-	[11]
CH5132799	14	6.7	56	120	500	36	-	[12]
Dual pan-isoform PI3K and mTOR inhibitors								
XL765	39	-	-	113	43	9	190/908	[14]
BEZ235	4	5.7	4.6	75	7	5	20.7	[16,141]
PF-04691502*	1.8	-	-	2.1	1.6	1.9	16	[142]
PF-05212384	0.4	0.6	0.8	6	8	6	1	[143]
GDC-0980	4.8	-	-	27	6.7	14	17	[144]
GSK2126458*	0.019	0.0078	0.0094	0.13	0.024	0.06	0.18/0.3	[18]
BGT-226	4	-	-	63	-	38	-	[24]
PI3Kα-specific inhibitors								
BYL719	5	4	5	1,156	290	250	> 9,100	[145]
PI3Kβ-specific inhibitors								
GSK2636771	-	-	-	5.2	58	-	-	[86]
PI3Kδ-specific inhibitors								
GS-1101 (CAL-101)	820	-	-	565	2.5	89	> 1,000	[46]
AMG319	-	-	-	-	< 10	-	-	[52]

A dash indicates no data available.

mTOR = mammalian target of rapamycin.

Ki*: binding affinity

**Table 3:** Summary of clinical activity of phosphatidylinositol 3-kinase (PI3K) inhibitors in phase I clinical trials

Agent/dose	N	Selected	Tumor type with PR (RECIST)	Molecular profile status	References
Pan-isoform PI3K inhibitors					
SAR245408 (XL147)	75	No	NSCLC	No alteration	[6]
BKM120	66	No (expansion only)	TN breast	KRAS mutation	[7]
			ER+ HER2- breast	PIK3CA mutation	
			Parotid cancer	PIK3CA mutation	
GDC-0941					
Once a day	42	No	Melanoma	BRAF V600E mutation	[8]
Once a day or twice a day	97	No	ER+, HER2- breast	Unknown	[9]
			Endocervical adenocarcinoma	PIK3CA mutation	
BAY 80-9646					
Dose escalation	17	No	None	-	[10]
Expansion: solid	30	No	ER+ HER2- Breast	No alteration	[11]
			ER+ HER2+ Breast	HER2 amplification	
Expansion: NHL	5	No	Follicular lymphoma (5 patients)	No alteration (0 of 5 patients)	[11]
CH5132799	31	No	None	-^a^	[12]
PX-866	84^b^	No	None	-	[13]
Pan-isoform PI3K-mTOR inhibitors					
SAR245409 (XL765)					
Solid tumors	83	No	None	-	[14]
Lymphoma	16	No	Mantle cell lymphoma	Unknown	[15]
			Transformed lymphoma	Unknown	
			Diffuse large B-cell lymphoma	Unknown	
BEZ235					
Once a day	59^c^	No (expansion only)	ER+ HER2- breast	Unknown	[16]
			NSCLC	PTEN mutation (Cowden)	
Twice a day	16	No	None	-	[17]
GSK2126458	129	No (expansion only)	Renal	No alteration	[18]
			Renal	PTEN loss	
			Bladder	PIK3CA mutation	
			Bladder	Unknown	
GDC-0980	42	No	Adrenal cortical	Unknown	[19]
	32	No	None	-	[20]
SF-1126	39	No	None	-	[21]
PF-04691502	33	No	None	-	[22]
PF-05212384	53	No (expansion only)	Ovarian cancer	No alteration	[23]
			NSCLC	EGFR mutation	
BGT-226	57	No	None	-	[24]
PI3Kα-specific inhibitors					
BYL719	35	Yes	ER+ breast	PIK3CA mutation	[25]
			Cervix	PIK3CA mutation	
			Colon	PIK3CA and KRAS mutations	
PI3Kδ-specific inhibitors					
GS-1101					
CLL	54	No	26% RR according to IWCLL	Unknown	[26]
Non-Hodgkin's lymphoma	49	No	Indolent NHL (15 PR out of 24); mantle cell lymphoma (10 PR out of 16)	Unknown	[27]

^a^Patient with ovarian cancer, with PIK3CA mutation still receiving treatment, with -17.2% reduction of target lesions and 75% decrease in CA-125.

^b^A total of 56 patients evaluable for response.

^c^A total of 51 patients evaluable for response.

BRAF = v-raf murine sarcoma viral oncogene homolog B1; CA = cancer antigen; CLL = chronic lymphocytic leukemia; EGFR = epidermal growth factor receptor; ER = estrogen receptor; HER2 = human epidermal growth factor receptor 2; IWCLL = International Workshop on Chronic Lymphocytic Leukemia; KRAS = v-Ki-ras2 Kirsten rat sarcoma viral oncogene homolog; mTOR = mammalian target of rapamycin; NHL = non-Hodgkin's lymphoma; NSCLC = non-small cell lung cancer; PIK3CA = phosphatidylinositol 3-kinase p110α isoform gene; PR = partial response; RECIST = Response Evaluation Criteria In Solid Tumors; RR = response rate; TN = triple negative.

## Strategies to optimize the development of PI3K inhibitors

The development of PI3K inhibitors is rapidly evolving with newer and more potent compounds entering clinical trials. Of particular interest are the isoform-specific PI3K inhibitors, which offer the potential of achieving greater selective target blockade while minimizing off-target effects due to inhibition of other isoforms as in the case of pan-PI3K inhibitors (Table 2). Whether these compounds may be superior to pan-PI3K inhibitors in safety and efficacy, and which patient populations may benefit the most from their use, are questions yet to be addressed. In addition, first-in-human studies of different PI3K inhibitors [6-27] have used variable approaches in patient inclusion ranging from unselected populations to restriction of patients with PI3K pathway alterations (Table 3). The results of these studies may help guide the design of future clinical trials. Patient selection can be enhanced through an improved understanding of the biological significance of PI3K pathway alterations in each tumor type and, even more specifically, in each patient. Lastly, the translation of antitumor activity observed in preclinical models to the clinical setting has been largely disappointing for PI3K inhibitors. As in the case of many other anticancer agents whereby proof of target inhibition in phase I trials is not straightforward, it is often uncertain if the dose ranges delivered in early trials of PI3K inhibitors can induce such effects at the tumoral level. Thus, there is a continued need whenever feasible to obtain tumor tissues during treatment for mechanistic proof of pathway engagement. Such pharmacodynamic data, together with relevant pharmacokinetic results, may help guide optimal dosing schedules. Tumor biopsy at disease progression among initial responders is also highly encouraged, in order to appreciate the underlying mechanisms of resistance and enable selection of the most appropriate therapy to overcome them.

## PI3K isoform-specific inhibitors

The PI3Ks are grouped into three classes (I, II and III) based on their structural characteristics and substrate specificity [2]. Class I PI3Ks are further divided into class IA enzymes, which include p110α, p110β and p110δ, while p110γ constitutes class IB [2]. In mammals, p110α and p110β are ubiquitous while p110γ and p110δ are expressed preferentially in leukocytes [28,29]. This distribution justifies the most relevant role of p110γ and p110δ in inflammatory diseases and the implication of p110δ in hematological malignancies. Class II PI3Ks seem to be implicated in exocytosis, cell migration, smooth muscle cell contraction, glucose metabolism and apoptosis [30]. Class III PI3Ks regulate cellular trafficking of vesicles and proteins [2]. Class I PI3Ks are involved in cell growth, survival and metabolism, therefore represent one of the most sought after targets in cancer therapeutics.

## PI3Kα-specific inhibitors

In addition to its effects on cell growth, proliferation and survival, class IA PI3K regulates glucose metabolism through insulin signaling [31-33]. It is commonly deregulated in cancer through mutations or amplifications of the *PIK3CA *gene or through alterations in the function of upstream tumor suppressors such as PTEN (Table 1). About 80% of the mutations of the *PIK3CA *gene are clustered in three hotspots in the *p110α *gene that encodes the catalytic subunit: two in the helical domain (E542K and E545K) and one in the kinase domain (H1047R) [34]. *PIK3CA *mutations are oncogenic per se, as they can induce the generation of tumors in several preclinical models without other molecular aberrations [35-37].

In addition to experiments in genetically engineered mice [31,32], the first generation of PI3Kα-specific inhibitors, while less isoform selective than the more recent compounds, have been instrumental in defining the biologic role of different PI3K isoforms in normal and cancer cells [33,38,39]. However, these agents have provided only inconclusive data on their antitumor activity in cell lines harboring *PIK3CA *mutations compared to those that are *PIK3CA *wild-type [40,41]. One of the main reasons is the limited number of cell lines in which these compounds have been evaluated. Cell lines without *PIK3CA *mutations often harbor alterations in oncogenic tyrosine kinase receptors, such as *ERBB2 *amplification, which preferentially uses the p110α isoform for signal transduction [32]. However, some of the cell lines harboring *PIK3CA *mutations had additional molecular aberrations, some of which are known mechanisms of resistance [41].

The new PI3Kα-isoform specific inhibitors have shown promising activity in cell lines harboring *PIK3CA *mutations [42,43]. In addition, the screening of one of these compounds, BYL719, in a large genomically characterized cell line panel, has revealed that besides *PIK3CA *mutations, the presence of *PIK3CA *amplification or *ERBB2 *amplification correlated with higher drug sensitivity. Conversely, *BRAF *and *PTEN *mutations were correlated with resistance. v-Ki-ras2 Kirsten rat sarcoma viral oncogene homolog (*KRAS*) mutation by itself was not associated with either sensitivity or resistance, although the coexistence of *KRAS *and *PIK3CA *mutations was usually associated with a lack of response [42].

Several new generation PI3Kα-selective inhibitors are currently being evaluated in phase I clinical trials, including BYL719 (NCT01219699), INK-1114 (NCT01449370) and GDC-0032 (NCT01296555). The clinical results of the dose escalation part of the phase I trial investigating BYL719 have recently been presented [25]. Trial enrollment was restricted to patients with solid tumors harboring *PIK3CA *mutations or amplifications. This population was selected based on the higher antitumor activity observed in preclinical models with *PIK3CA *mutations or amplifications using the Cancer Cell Line Encyclopedia [42]. This was the first reported study of a PI3K inhibitor in which molecular prescreening was undertaken starting from the dose escalation part. A total of 35 patients have been enrolled thus far and the maximum tolerated dose has been determined as 400 mg orally on a continuous once daily schedule. Three patients, all of whom received doses ≥ 270 mg/day, have achieved a partial response. The tumor types of these responders were estrogen receptor positive breast cancer, cervical cancer and *KRAS*-mutant colon cancer, and *PIK3CA *mutations were detected in all three cases (E542K/V, E545K and R88Q respectively). In addition, prolonged disease stabilization, defined as that lasting for ≥ 4 months, has been observed in ten patients with primary tumor sites from oral cavity, salivary gland, colon, and estrogen receptor positive breast. Among them, five patients have remained on study treatment for more than 6 months [25]. The clinical response observed in the colon cancer patient with coexistent *KRAS *and *PIK3CA *mutations contrasts with the preclinical finding in which such coexpression generally conferred resistance to BYL719. Tumor heterogeneity may partly explain the clinical results, if for instance, these mutations are not coexistent in all geographic areas, or if the two mutations have different tumor-driving functions. Furthermore, this case illustrates the molecular complexities in human malignancies that often cannot be reliably reflected by preclinical models.

From a safety perspective, the most commonly observed adverse effects associated with BYL719 were hyperglycemia, nausea, fatigue, rash and gastrointestinal toxicities [25], all of which are also frequently encountered with the pan-PI3K inhibitors. Although the spectrum of toxicities encountered between BYL719 and the pan-PI3K inhibitors are similar, hyperglycemia represents the most frequent and dose-limiting adverse event with BYL719. Given the interaction between PI3K pathway inhibition and insulin signaling, occurrence of this on-target toxicity supports proof-of-mechanism. A relevant question is whether an isoform-selective PI3K inhibitor is able to achieve greater target inhibition than the pan-PI3K inhibitors while producing a similar degree and extent of side effects. At present, there is a paucity of published preclinical data comparing any of the PI3Kα-selective inhibitors currently in clinical development with pan-isoform PI3K inhibitors. While early results from the phase I trial of BYL719 appear encouraging, direct comparison of the preliminary efficacy results achieved with this agent against those reported with the pan-isoform PI3K inhibitors would be invalid, as none of the early phase trials involving pan-PI3K inhibitors have been specifically designed to evaluate only the *PIK3CA *mutant population. Even among those cases which utilized an enrichment strategy in the expansion cohort to select for patients with molecular alteration in the PI3K pathway, a variety of alterations such as *PIK3CA *mutation or amplification, *PTEN *mutation or loss of PTEN expression have been included (Table 3).

## PI3Kδ-specific inhibitors

In contrast to the ubiquitously expressed p110α and p110β isoforms, p110δ is mainly expressed in leukocytes [28,29]. Its overexpression has been observed in a wide range of lymphoproliferative disorders including chronic lymphocytic leukemia (CLL) [44], multiple myeloma [45], diffuse large B-cell lymphoma [46], B-cell acute lymphoblastic leukemia [46], follicular lymphoma [46], mantle cell lymphoma [47,48], and Hodgkin's lymphoma [49].

Currently, two PI3Kδ-specific inhibitors are in clinical development: GS-1101, previously known as CAL-101, and AMG 319. GS-1101 has shown preclinical activity as a single agent against different lymphoid malignancies including CLL [44,46], multiple myeloma [45], mantle cell lymphoma [47], Hodgkin's lymphoma [49] and B-cell acute lymphoblastic leukemia [46]. GS-1101 has been shown to partially revert stroma-induced resistance to conventional cytotoxic drugs in CLL [50,51]. In addition, synergy with targeted therapies such as the mammalian target of rapamycin (mTOR) inhibitor everolimus [47] or the proteasome inhibitor bortezomib [45], has been described in mantle cell lymphoma and multiple myeloma, respectively. AMG 319 has shown activity against several cell lines derived from B-cell malignancies [52], and synergy with vincristine in diffuse B-cell lymphoma has been observed [52,53].

Among the PI3Kδ-specific inhibitors, clinical data have been published thus far only with GS-1101. Early signs of antitumor activity were found in the phase I clinical trial in selected relapsed or refractory hematologic malignancies including patients with CLL and non-Hodgkin's lymphoma [54,55]. The most recent report has shown that in 80% of the 54 patients with CLL enrolled in the phase I trial, ≥ 50% lymphadenopathy shrinkage was observed and the overall intention-to-treat response rate by the 2008 International Workshop on Chronic Lymphocytic Leukemia (IWCLL) response criteria [56] was 26%. The most relevant grade 3 or higher adverse events were pneumonia, neutropenia (7% of patients developed febrile neutropenia), thrombocytopenia, anemia, and transaminase elevation [26]. In patients with non-Hodgkin's lymphoma, 15 out of 24 patients with indolent non-Hodgkin's lymphoma and 10 out of 16 patients with mantle cell lymphoma achieved a partial response. However, none of the nine patients with diffuse large B-cell lymphoma had a partial response. The observed serious toxicities (grade ≥ 3) were similar to those reported in the CLL arm, which included hematological toxicities and transaminase elevation [27]. Based on the striking monotherapy activity observed in these two population groups, GS-1101 is being evaluated in a phase I clinical trial in combination with several compounds active in hematological malignancies. The data of the combination arms of GS-1101 with rituximab with or without bendamustine [57,58] and in combination with ofatumumab [59] have been recently presented. Substantial antitumor activity has been described with both regimens with the expected toxicities based on the single agent toxicity profile. Results from the fludarabine, chlorambucil, everolimus and bortezomib arms have not been presented yet (NCT01088048). There are ongoing phase III clinical trials for patients with CLL investigating the combination of GS-1101 with rituximab (NCT01539512), and with rituximab and bendamustine (NCT01569295). Besides GS-1101, AMG 319 is another PI3Kδ-specific inhibitor in clinical development, a phase I clinical trial of this agent in patients with relapsed or refractory lymphoid malignancies is ongoing (NCT01300026).

The question of whether PI3Kδ-specific or pan-isoform PI3K inhibition constitutes a more optimal therapeutic strategy in patients with lymphoid malignancies is still under debate. Preclinically, some pan-isoform PI3K inhibitors have shown signs of activity in selected lymphomas and CLL [48,60-63]. In certain lymphoma subtypes, the activity of the pan-isoform PI3K inhibitors GDC-0941 and SF-1126 could potentially be superior [48,62,63].

In the clinical setting, the pan-isoform PI3K inhibitor SAR245408 (XL147) [64] and the pan-isoform PI3K and mTOR inhibitor SAR245409 (XL765) [15] have been evaluated in patients with lymphoma, as an expansion cohort of the respective phase I clinical trials. Observed grade 3 or higher adverse events with SAR245408 have been primarily hematological toxicities including neutropenia and thrombocytopenia, as well as hyperglycemia [64]. Hyperglycemia was not commonly reported with SAR245409, but grade 3 transaminase elevation was observed in 2 out of 15 patients [15]. The antitumor activity of SAR245408 has not yet been reported [64]. Among the 13 patients with lymphoma treated in the phase I clinical trial with SAR245409, 3 patients (mantle cell lymphoma, transformed lymphoma and diffuse large B-cell lymphoma) achieved a partial response [15]. SAR245409 is currently being evaluated as a single agent in a phase II clinical trial in patients with selected types of lymphoma or leukemia (NCT01403636) and in a phase I trial in combination with bendamustine with or without rituximab (NCT01410513).

## PI3Kβ-specific inhibitors: the role of PTEN alteration

The signaling of the PI3Kβ isoform is mediated via GPCR [65-71] while the PI3Kα isoform preferentially mediates via RTK, however, platelet-derived growth factor receptor is able to sustain its signaling through the PI3Kβ isoform in the absence of the PI3Kα isoform [32].

The PI3Kβ isoform is oncogenic when deregulated [65]. There are no *PIK3CB *mutations described in cancer so far. The most common event that leads to PI3Kβ-isoform signaling deregulation is PTEN deficiency, although *PIK3CB *amplification has been described in breast cancer [72]. PTEN is a lipid phosphatase that dephosphorylates the 3-phosphoinositide products of PI3K [73]. PTEN deficiency is a frequent event in cancer [74] (Table 1), which can occur through several mechanisms including *PTEN *mutation, *PTEN *deletion, epigenetic changes [75-79], miRNA-mediated regulation [80-82] or post-translational modifications [83,84].

In preclinical models, it has been demonstrated that PTEN-deficient tumors depend on the PI3Kβ isoform for pathway activation, growth and survival [65,85]. The preclinical activity of several PI3Kβ-specific inhibitors in PTEN-deficient cell lines and xenograft models has been recently communicated [86-88]. In the clinical setting, a phase I clinical trial with the selective PI3Kβ-selective inhibitor GSK2636771 in patients with advanced solid tumors with PTEN deficiency is currently ongoing (NCT01458067), and a phase I clinical trial with the PI3Kβ-selective inhibitor (SAR260301) in solid tumors as a single agent and in combination with vemurafenib in BRAF mutant melanoma, has recently been initiated (NCT01673737).

## Patient selection

One of the major challenges in the clinical development of PI3K inhibitors is to identify the appropriate patient populations most likely to benefit from the treatment. In the current era where many drug targets are entering clinical evaluation and even more compounds are being developed to interrogate such targets, a rational approach is to intensify biomarker research in the preclinical setting and then incorporate them in early phase clinical trials. Both pharmacodynamic markers to prove biological effect and predictive biomarkers to identify sensitive or resistant populations are of interest, and their exploration in valid preclinical models would inform clinical development.

In preclinical models, cell lines harboring *PIK3CA *mutation, or amplification of *PIK3CA *or *ERBB*2 have shown sensitivity to different PI3K inhibitors, including pan-isoform PI3K inhibitors [89-91] or PI3Kα-specific inhibitors [42,43]. However, the role of PTEN loss as a predictor of responsiveness to PI3K inhibitors is less clear [40,90,92,93]. In the clinical setting, the retrospective analysis of 217 patients referred to the MD Anderson Cancer Center revealed that those with *PIK3CA *mutant tumors treated with PI3K-AKT-mTOR axis inhibitors demonstrated a higher objective response rate than patients without such mutations [94,95]. However, the majority of these patients received combination therapies that included an mTOR inhibitor, and not a PI3K inhibitor. In addition, there are inherent biases to retrospective analyses, and these results should be considered exploratory and interpreted cautiously.

As depicted in Table 3, initially phase I clinical trials with PI3K inhibitors have been developed in unselected patient populations. As preclinical data of sensitivity to pan-PI3K inhibitors in tumors harboring relevant molecular aberrations become available [89-91], different enrichment strategies have been adopted. These strategies range from the selection of patients with any PI3K pathway alterations in the expansion cohort of phase I trials, to the approach utilized in the recent phase I trial of the PI3Kα-specific inhibitor BYL719 in which only patients with *PIK3CA *mutations or amplifications were enrolled. It is invalid to make a direct comparison between unselected versus selected approaches for patient recruitment, as other factors, such as the anticancer activity of each compound, the number of patients treated at suboptimal doses, pharmacokinetic issues, or the presence of different molecular events that can modify the sensitivity to PI3K inhibitors (such as *KRAS *mutations), can be confounding. However, preliminary experience from the phase I trial of BYL719 suggests that it is reasonable to select patients based on specific molecular aberrations which are justified by appropriate preclinical models. Importantly, this study has performed large scale screening in local institutions to identify patients with uncommon molecular characteristics without compromising timely enrollment, a finding that supports the feasibility of molecular prescreening already implemented by many large drug development programs [96,97].

## Elucidation of mechanisms of pathway activation and resistance

Results from the first clinical trials (Table 3) of various PI3K inhibitors may shed insight to help identify tumors in which these agents exert sufficient activity to inactivate the PI3K pathway. Unlike BRAF or ALK inhibitors that have demonstrated very early on in their development anticancer activity against patient populations whose tumors are uniquely sensitive to these agents, objective responses seen in the early clinical trials of PI3K inhibitors were less predictable. While some of the responders had PI3K pathway aberrant tumors, there were many who did not respond despite harboring relevant molecular features, as well as others who responded without obvious molecular predisposition. There is clearly a context dependence in which tumor histology may be relevant, as the functionality of the same genomic aberration across different tumor types may vary. However, histology is unlikely the only context as patients with the same tumor type harboring similar molecular aberrations often have different outcomes despite receiving the same matched therapy [98]. A key challenge in the clinical evaluation of PI3K inhibitors is to differentiate patients whose tumors are addicted, dependent, versus resistant, to a PI3K isoform [99].

PI3K isoform-addicted tumors correspond to those in which a dramatic and sustained response is observed with PI3K inhibitors. These tumors may be so vulnerable that even partial pathway inhibition is sufficient to lead to clinical responses. These patients may be extraordinary candidates for treatment with PI3K isoform-selective inhibitors to achieve a high therapeutic index by minimizing off-target adverse effects while obtaining adequate target inhibition.

PI3K-dependent tumors are those which likely require a complete or near complete pathway inhibition to achieve meaningful responses. Tumors which may belong to this categorization include those with upstream RTK hyperactivation, those with simultaneous activation of several points along the PI3K pathway [100,101], those harboring oncogenic events which can signal through different isoforms [32,102], or those which were initially addicted to an isoform but have acquired resistance to reactivate the PI3K pathway via alternate mechanisms [99]. Breast cancers with simultaneous human epidermal growth factor receptor 2 (HER2) amplification and *PIK3CA *mutation represent good examples of simultaneous RTK hyperactivity and activation of the PI3K pathway at several levels [100,101]. Interesting activity has been reported in the clinical setting with the combination of the PI3K-mTOR inhibitor BEZ235 and trastuzumab, presumably due to the effect of sufficient modulation of both mitogen-activated protein kinase (MAPK) and PI3K pathways [98]. In a preclinical experiment, Liu *et al*. constructed a PI3K isoform-addicted mouse model of breast cancer conditionally expressing *PIK3CA^H1047R^*. Tumor response was observed after suppressing *PIK3CA^H1047R ^*expression, but spontaneous tumor recurrence was detected in some animals after initial response. Such tumors appeared to have escaped oncogenic addiction and either remained dependent on the PI3K pathway and respond to the pan-isoform PI3K inhibitor GDC-0941 or became totally resistant, with the amplification of *c-MET *and *c-MYC *being implicated in these evolutions, respectively [99]. To translate these findings to the clinic, it would be informative to perform tumor biopsies at the time of disease progression in patients who have initially responded to PI3K inhibitors, to determine if the tumor remains dependent on the PI3K pathway and thus may benefit from combinatorial strategies, or whether it has developed resistance through an independent mechanism.

The last group is constituted by those tumors deemed to be resistant to PI3K inhibition, such that interrogation of the PI3K axis alone will be unlikely to yield any clinical benefit. This molecular behavior could either be due to a primary *de novo *resistance [103] or an acquired resistance after the selective pressure of PI3K inhibition [101]. It is important to recognize resistant subtypes early on in the disease course, as some cases might be appropriate candidates for combination treatment, such as simultaneous inhibition of the PI3K and MAPK pathways [103]. The ability to distinguish various molecular alterations in tumors and their translation to unique biological behaviors would enable a more effective strategy to individualize treatment with PI3K inhibitors.

## Therapeutic targeting of the PI3K pathway

The decision of whether PI3K isoform-selective inhibitors are more therapeutically appealing than pan-PI3K inhibitors awaits the maturation of results from ongoing clinical trials. In addition, other challenging questions remain in the clinical development of PI3K inhibitors. For instance, the most optimal drug administration schedule for PI3K inhibition remains elusive. Preclinical models are needed to investigate dosing schedules in tumors which are addicted, dependent, versus resistant to PI3K inhibition to decipher how best to effectively modulate the pathway in each scenario. Dosing schedules may range from the administration of intermittent high doses to completely abrogate the pathway versus continuous low doses to provide sustained but less intense inhibition of the pathway. The availability of both intravenous and oral pan-isoform PI3K inhibitors enables the evaluation of the efficacy and toxicity of this class of agents using different administration schedules. In addition, recent preclinical work has highlighted schedule dependence when combining two different anticancer drugs [104], the relevance of this phenomenon to combinations involving PI3K inhibitors is yet to be assessed. Some early phase trials are evaluating this question in the clinical setting, such as the recently presented study investigating different schedules of the pan-PI3K inhibitor BKM120 in combination with letrozole [105].

Given the lack of significant single agent activity with PI3K inhibitors in many patients tested so far on clinical trials, it is likely that combinatorial approaches incorporating PI3K inhibitors are necessary to achieve meaningful therapeutic effects. Activation of PI3K pathway has been described as a mechanism of resistance to hormone therapy and anti-HER2 therapy in breast cancer [100], clinical trials of combinations of these agents with PI3K inhibitors are currently ongoing. However, *KRAS *mutation has been described as a resistant factor for PI3K inhibitors, through its activation of the MAPK pathway. Thus, several targeted combination trials of PI3K inhibitors and mitogen-activated protein kinase kinase (MEK) inhibitors are underway in the clinic. However, some *KRAS *mutations preferentially signal through the PI3K pathway [106], this may explain the partial response observed with BKM120 in a patient with triple negative breast cancer whose tumor harbored a *KRAS *mutation. A further limitation to finding the most appropriate targeted combination is the inability to readily decipher whether molecular alterations detected represent driver events. Tumor heterogeneity contributes an additional layer of complexity in the selection of targeted combinations [107].

Despite therapeutic advances that have now rendered PI3K a druggable target, many questions remain unanswered. Are alternate pathway activation and tumor heterogeneity the reasons why PI3K inhibitors are not declared as panacea based on the currently available clinical data? Is the pathway so critical in the human organism that compensatory feedback mechanisms emerge very quickly upon inhibition? Are existent PI3K inhibitors in clinical development potent enough with optimal pharmacokinetic and pharmacodynamic properties? Would the early phase clinical results have been superior if all patients had been preselected according to molecular characteristics? As knowledge accumulates in the PI3K pathway and more potent PI3K inhibitors become available, rational application of these agents as monotherapy or in combination is within reach.

## Conclusions

Isoform-specific PI3K inhibitors are now entering clinical development; they appear promising by proposing to achieve a greater degree of isoform inhibition with fewer off-target side effects. Tumors differ in their response thresholds to PI3K inhibitors based on their degree of addiction, dependence or resistance to this oncogenic pathway. Characterization of somatic molecular alterations and integration of this information into the treatment algorithm may enable more effective therapeutic targeting using PI3K inhibitors. It is plausible that the best clinical results could only be achieved by deepening the biological knowledge of how each individual tumor would behave upon PI3K pathway interrogation. Only in that context can one most appropriately select the best agent, either as monotherapy or in combination, to administer using the most effective dosing schedule.

## Abbreviations

ALK: anaplastic lymphoma receptor tyrosine kinase; BRAF: v-raf murine sarcoma viral oncogene homolog B1; CLL: chronic lymphocytic leukemia; EML4: echinoderm microtubule-associated protein-like 4; GPCR: G protein-coupled receptor; KRAS: v-Ki-ras2 Kirsten rat sarcoma viral oncogene homolog; MAPK: mitogen-activated protein kinase; MEK: mitogen-activated protein kinase (MAPK) kinase; PI3K: phosphatidylinositol 3-kinase; PIK3CA: phosphatidylinositol 3-kinase p110α isoform gene; PIP2: phosphatidylinositol 4,5-bisphosphate; PIP3: phosphatidylinositol 3,4,5-trisphosphate; PTEN: phosphatase and tensin homologue deleted on chromosome 10; TKR: tyrosine kinase receptor.

## Competing interests

LLS: research support from Novartis, GSK, Bristol-Myers Squibb, Pfizer, Genentech/Roche.

## Authors' contributions

IB and LLS: conception and design; manuscript writing; final approval of manuscript.

## Authors' information

IB is a clinical research fellow at the Drug Development Program at the Princess Margaret Cancer Center. The focus of her research is the evaluation of new investigational drugs in early phases of clinical development, as well as preclinical evaluation of combination of targeted agents, with a special focus on PI3K inhibitors. LLS is a Professor of Medicine and a Senior Medical Oncologist at Princess Margaret Cancer Centre. She holds a Cancer Care Ontario Chair in Experimental Therapeutics. Her research focus is in the area of new drug development and cancer genomics.

## Pre-publication history

The pre-publication history for this paper can be accessed here:

http://www.biomedcentral.com/1741-7015/10/161/prepub
